# Natural infection of *Lutzomyia longipalpis* (Cembrene-1 population) with *Leishmania infantum* in a new visceral leishmaniasis focus in the eastern region of São Paulo State, Brazil.

**DOI:** 10.1590/0037-8682-0586-2020

**Published:** 2021-02-26

**Authors:** Mariana Dantas da Silva, Fredy Galvis-Ovallos, Claudio Casanova, Vanessa Gusmon da Silva, João Augusto Franco Leonel, Trícia Maria Ferreira de Sousa Oliveira, Eunice Aparecida Bianchi Galati

**Affiliations:** 1 Universidade de São Paulo, Faculdade de Saúde Pública, Programa de Pós-Graduação em Saúde Pública, São Paulo, SP, Brasil.; 2 Universidade São Paulo, Faculdade de Saúde Pública, Departamento de Epidemiologia, São Paulo, SP, Brasil.; 3 Secretaria de Estado da Saúde, Superintendência de Controle de Endemias, Mogi Guaçu, SP, Brasil.; 4 Universidade de São Paulo, Faculdade de Medicina Veterinária e Zootecnia, Programa de Pós-Graduação em Epidemiologia Experimental Aplicado a Zoonoses, Pirassununga, SP, Brasil.

**Keywords:** *Lutzomyia longipalpis* complex, Cembrene-1, Natural infection, Visceral leishmaniasis

## Abstract

**INTRODUCTION:**

Visceral leishmaniasis (VL) transmission has been associated with two different populations of the *Lutzomyia longipalpis* complex in São Paulo state.

**METHODS:**

In a recent focus of VL, we captured and dissected sand flies and investigated *Leishmania infantum* infection by parasitological, PCR, and sequencing analysis.

**RESULTS:**

Flagellates were observed in 2 of 47 (4.2%) cembrene-1 *Lu. longipalpis* females. The sequences obtained matched those of *Le. infantum*.

**CONCLUSIONS:**

We found that the transmission of *Le. infantum* by cembrene-1 females may occur at a high rate in this focus of VL and presented new data on the vector capacity of this population.

Leishmaniases are caused by protozoa of the genus *Leishmania* (Trypanosomatidae), which are transmitted to vertebrates through the bite of infected females of phlebotomine sand flies (Diptera: Psychodidae). They constitute a complex of diseases with different clinical spectra, including cutaneous, mucocutaneous, and visceral forms, which may vary according to the *Leishmania* species involved and the host’s immune status, among other factors. Leishmaniases have been registered in 98 countries in tropical and subtropical regions, with estimates of 350 million people living in areas at risk of transmission^1^. From 2000 to 2018, a total of 64,463 new cases of visceral leishmaniasis (VL) with 4,476 deaths were recorded in Brazil. Most cases have been registered in the northeastern region (54%), followed by the northern (17%) and southeastern (16%) regions^2^. In São Paulo state (SP), from 1999 to 2019, 3,046 VL cases in humans with 267 deaths were recorded^3^, and in 178 of its 645 municipalities (27.6%), the presence of *Lutzomyia longipalpis*, the main vector of *Le. infantum* in Brazil, has been registered^4^.

The first record of the presence of vector *Lu. longipalpis* in an urban area in the state of São Paulo occurred in the western region, in the municipality of Araçatuba in 1997^5^, where the first canine and human autochthonous cases of visceral leishmaniasis were recorded in 1998 and 1999^6^, respectively. *Lutzomyia longipalpis* is considered a complex of species with four distinct populations present in Brazil, which are classified according to the type of pheromone produced^7^ ^,^ ^8^. Two of them, (*S*)-9 methylgermacrene-B and cembrene-1, occur in São Paulo with allopatric distribution. The (*S*)-9-methylgermacrene-B population occurs in urban areas of the municipalities of the western region, where it is associated with the presence of canine and human cases of VL, and its role as vector is well established. In contrast, the cembrene-1 population occurs mainly in rural areas of municipalities of the eastern region, where only canine visceral leishmaniasis (CVL) cases have been reported, and the parameters of its vector capacity need to be evaluated^7^ ^,^ ^8^_,_ including natural infection by *Le. infantum*. The presence of the cembrene-1 population has been observed in Valinhos municipality, in the eastern region of SP^7^ where autochthonous CVL transmission has been confirmed^4^; thus, the present study sought to investigate natural infection by *Le. infantum* in females of *Lu. longipalpis* in an area with CVL transmission in this municipality.

Study area: Valinhos belongs to the Atlantic Forest Biome. The municipality, with an area of 148,538 km^2^ and an estimated population of 124,024 inhabitants, is located in the eastern region of São Paulo state (22° 58'14″ S; 46° 59' 45″ W) in the Campinas Metropolitan Region. Its climate, according to the Köopen-Geiger classification, is subtropical humid^9^, with an average annual temperature of 21 °C; the rains are irregularly distributed throughout the year, with a humid summer from October to March and a dry winter from April to September^10^.

Two collections were undertaken in December 2018 on a housing estate located in the Nova Suiça neighborhood, where two CVL cases had previously been reported.

Three houses at a distance of 10 m, 40 m, and 200 m between the peridomicile and chicken houses were sampled. In the three chicken coops, a manual capture with a Castro aspirator was undertaken between 18.00 and 22.00 hours, and CDC light traps were installed between 18.00 and 08.00 hours. The collected specimens were transported to the FSP/USP Public Health Entomology Laboratory, where the females were dissected in a drop of phosphate buffered saline (PBS) and the gut was removed under stereoscopy and covered with a coverslip for observation by optical microscopy at 400× magnification. Females without flagellates in their guts were grouped in pools according to the date and place of capture. Females carrying flagellates were stored individually in microtubes containing isopropyl alcohol. Samples were analyzed for the identification of *Leishmania* spp. by polymerase chain reaction (PCR) and sequencing.

Sand fly DNA extraction was performed according to the protocol of BRUFORD et al*.* ^11^, as described by Galvis-Ovallos et al^8^. *Leishmania* spp. were detected using primers that amplify the conserved region of the kDNA minicircle 13A/13B, as described by Rodgers et al^12^. The positive samples from the previous PCR analysis were subjected to PCR with primers that amplify the internal transcribed spacer 1 (ITS-1) region of the rDNA, as described by El Tai et al^13^. As a positive control, the DNA of *Leishmania amazonensis* was used for the PCR analysis of *Leishmania* spp*.* infection. For all reactions, autoclaved ultrapure water was used as a negative control. Finally, the amplified products were subjected to 1.5% agarose gel electrophoresis (2% ITS-1 gene) (13A/13B) and stained with SYBER safe (Invitrogen) at 100 V for 60 min. Sequencing for identifying *Leishmania* spp*.* was performed only on the samples of those females confirmed as infected through parasitological and molecular examination*.* The amplifiers were purified with a commercial Illustra GFX PCR DNA and Gel Band Purification kit (GE Healthcare) and sent to the DNA Sequencing Service of the Human Genome and Stem Cell Research Center-IB-USP. The electropherograms were analyzed using the Chromas and BioEdit software. The sequences were then compared to those of the BLAST program (GenBank).

A female of *Migonemyia migonei* and two of *Pintomyia fischeri* were dissected; however, no flagellates were observed*.* A total of 47 *Lu. longipalpis* females were captured and dissected, and the presence of flagellates was observed in the gut of two of them (Figure 1). The two positive samples were stored individually, and the remaining 45 were grouped into four pools. The presence of *Le. infantum* was corroborated through DNA fragment amplification and sequencing in the two samples found to harbor flagellates (Figure 2). In the pools constituted by the females with negative results for the presence of flagellates in the dissection, no DNA of *Leishmania* spp. was detected. Therefore, a natural infection rate of 4.2% (2/47) was estimated by parasitological and molecular examination. In the two females infected with *Le. infantum*, a massive parasite infection with metacyclic forms was located in the thoracic digestive tract posterior to the stomodeal valve Figure 1A, Supplementary Movie 1, available in the following link: 

https://youtu.be/Oux12-VpZbY

**FIGURE 1:**
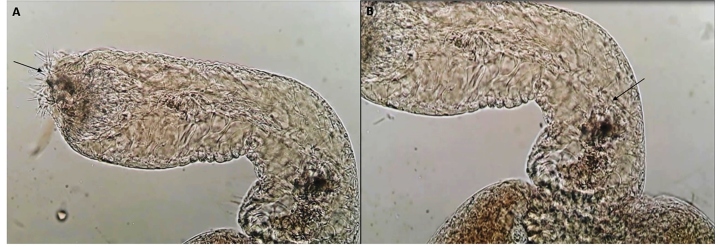
Presence of flagellated clusters (indicated by the arrow) in the parasitological exam positive for *Leishmania infantum*. **(A)** Region of the stomodeal valve. **(B)** Region of the thoracic midgut.

**FIGURE 2:**
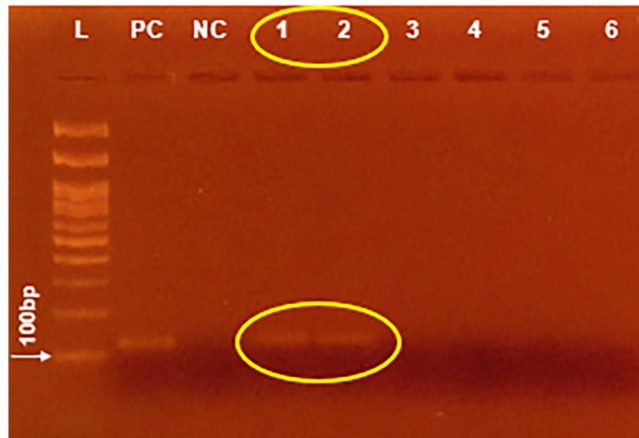
Agarose (2%) electrophoresis gel for *Leishmania*. spp. kDNA (120 base pairs) from *Lutzomyia longipalpis* females (1-6) with 100 bp molecular weight marker (KASVI) (L), *Leishmania amazonensis* (IFLA/BR/1967/pH8) DNA (positive control) (PC) and ultrapure water (negative control) (NC).

The identification of females of *Lu. longipalpis* naturally infected with *Le. infantum* in the municipality of Valinhos highlights the participation of the cembrene-1 population of *Lu. longipalpis* complex in the VL agent transmission cycle in São Paulo state. In addition, the infection rate of 4.2% (2/47), together with a local CVL prevalence of 20% (CASANOVA 2019, personal information), points to a high risk of transmission to the human population. Infection rates of up to 19% have been described, and these values are obtained through molecular techniques, owing to the greater sensitivity of these techniques^8^ ^,^ ^14^; however, in the present investigation, both techniques used showed the same sensitivity. Although natural infection rates may vary in different foci depending on the detection method employed (parasitological or molecular), as well as on the prevalence of reservoirs, the observation of flagellates by dissection permitted the identification of important characteristics such as the intensity of the infection and the localization of the parasites in the sand fly gut. The high infection rate observed in females of *Lu. longipalpis* (cembrene-1) and the massive parasite load in their gut emphasize the importance of entomological surveillance in the locality sampled. Although sensitivity in the dissection technique used is influenced by the observer's experience and skills and it is impossible to identify the parasite species involved only on the basis of its morphology^15^, combination of the technique with molecular methods constitutes an important epidemiological tool for monitoring parasites in vector populations. The presence of massive parasite infection provides evidence that allows us to draw inferences regarding the vector capacity of this population. Thus, despite the small and restricted sand fly samples (47 females, from a single site) available for dissection, this study showed that the transmission of the parasite by females of the cembrene-1 *Lu. longipalpis* populations may occur at a high rate (4.2%) in the presence of a high infection level in reservoirs (20%). This evidence highlights the need for entomological surveillance for implementing prevention and control measures to reduce the risk of transmission to the human population, as well as in other areas in the eastern region of São Paulo state where this sand fly population occurs.
